# Endobronchial Perfluorocarbon Reduces Inflammatory Activity before and after Lung Transplantation in an Animal Experimental Model

**DOI:** 10.1155/2013/193484

**Published:** 2013-12-26

**Authors:** Luiz Alberto Forgiarini Junior, Arthur Rodrigo Ronconi Holand, Luiz Felipe Forgiarini, Darlan Pase da Rosa, Norma Anair Possa Marroni, Paulo Francisco Guerreiro Cardoso, Cristiano Feijó Andrade

**Affiliations:** ^1^Postgraduate Program in Pulmonology, Federal University of Rio Grande do Sul (UFRGS), 90040-060 Porto Alegre, RS, Brazil; ^2^Methodist University (IPA), 906301-170 Porto Alegre, RS, Brazil; ^3^Thoracic Surgery Department, Laboratory of Airways and Lung, Hospital de Clínicas de Porto Alegre (HCPA), Ramiro Barcelos 2.350, 90035-903 Porto Alegre, RS, Brazil; ^4^Lutheran University of Brazil (ULBRA), 92425-900 Canoas, RS, Brazil; ^5^Department of Cardiopneumology, Division of Thoracic Surgery, Laboratory of Medical Investigation (LIM 61), Heart Institute (InCor), Hospital das Clínicas, Faculdade de Medicina da Universidade de São Paulo, 05403-000 São Paulo, SP, Brazil; ^6^Santo Antônio Children's Hospital, 90020-090 Porto Alegre, RS, Brazil

## Abstract

*Background*. The aim of this study was to evaluate the use of liquid perfluorocarbon (PFC) as an adjuvant substance for lung preservation and assess its role in pulmonary protection after transplantation. *Methods*. Seventy-two rat lungs were flushed with low-potassium dextran (LPD) solution and randomized into three main groups: control with LPD alone and experimental with 3 (PFC3) and 7 mL/kg (PFC7) of endobronchial PFC instilled just after harvest. Each group was divided into four subgroups according to preservation time (3, 6, 12, and 24 hours). Afterwards, we performed lung transplantation using rat lungs preserved for 12 hours with LPD alone or with 7 mL/kg of endobronchial PFC. *Results*. There was a significant increase in oxidative stress in the control group at 6 h of cold ischemic time compared with the PFC3 and PFC7 groups. The apoptotic activity and NF-*κ*B expression were significantly higher in the control group compared with the PFC groups at 3, 12, and 24 h of cold preservation. After transplantation, the NF-*κ*B, iNOS, and nitrotyrosine expression as well as caspase 3 activity were significantly lower in the PFC groups. *Conclusion*. The use of endobronchial PFC as an adjuvant to the current preservation strategy improved graft viability.

## 1. Introduction

Lung transplantation is an established therapy for patients with end-stage lung disease [1]. In the early postoperative period, recipients may present with severe complications such as ischemia-reperfusion (IR) injury, acute rejection, and infection [2]. IR injury affects up to 20% of patients following lung transplantation and may lead to severe primary graft dysfunction, increasing morbidity/mortality [3].

Severe primary graft dysfunction is the end result of a series of events occurring from the time of brain death to the time of lung reperfusion after transplantation [4]. Strategies to prevent lung dysfunction resulting from IR injury have been focused on the selective assessment of donor lungs [5], effective techniques of lung preservation [6], and careful management of transplanted lungs after reperfusion [7].

The early stage of reperfusion injury following lung transplantation is initially mediated by the innate immune response through the activation of alveolar macrophages in the lung donor with the consequent production of proinflammatory cytokines that exacerbate the inflammatory response, leading to cell death through an apoptotic pathway [8–12]. Longer ischemic periods result in an escalating proportion of cell death, resulting in a significant derangement in lung function, which suggests a positive correlation between preservation time and organ performance [13]. This inflammatory response and induction of apoptosis may be mediated by nuclear transcription factor kappa B (NF-*κ*B), which would trigger the posttransplant lung injury and cell death pathways [8, 14].

Numerous studies have been performed to optimize the technique of lung preservation [15–19]. Lung retrieval for transplantation includes the flushing of the lungs with a hypothermic preservation solution to decrease its metabolic rate and energy requirements during storage [20]. Although hypothermia remains an essential component of the organ preservation strategy, it is, nevertheless, associated with a series of events that may induce the upregulation of molecules on the cell surface membrane and proinflammatory mediators [21].

Liquid perfluorocarbons (PFCs) administered endobronchially have been used in different models of lung injury [22, 23], primarily as an alternative method of lung ventilation, known as partial liquid ventilation [19]. PFCs wash out alveoli debris, recruit collapsed alveoli, improve gas exchange, protect pulmonary architecture and have anti-inflammatory and antioxidant properties [24, 25]. There is a limited number of studies using endobronchial PFCs in the setting of lung transplantation, with conflicting results [19, 22, 26].

The objective of this study is to evaluate the use of liquid PFC as an adjuvant for lung preservation and its role as a lung protective substance after transplantation. The study was designed to test the impact of different PFC doses for different preservation periods and evaluate the effects of PFC on cell death, the inflammatory response, and oxidative stress in the transplanted lung grafts.

## 2. Methods

The protocols for all experiments involving animal surgery were approved by the Research Ethics Committee of the Hospital de Clinicas de Porto Alegre. All animals received humane care in accordance with the National Research Council (Guide for the Care and Use of Laboratory Animals, NIH revised 19). Male Wistar rats from the hospital's central animal laboratory weighing between 250 and 300 g were used for all animal experiments.

### 2.1. PFC Dose versus Preservation Time Studies

To define the amount of PFC administered for endobronchial lung preservation, we evaluated three different dose regimens. Initially, we tested 3, 7, and 15 mL/kg body weight of PFC given endobronchially in a pilot study. Twenty male Wistar rats were divided into four groups (*n* = 5) according to the PFC dose. One control group did not use PFC. The animals were anesthetized (ketamine 100 mg/kg and xylazine 15 mg/kg, administered intraperitoneally) and ventilated with a tidal volume of 8 mL/kg of body weight, a respiratory rate of 70–80 breaths/min, a fraction of inspired oxygen of 0.2 (room air), and a positive end expiratory pressure of 2 cm H_2_O (Harvard Rodent Ventilator, model 683; Harvard Apparatus Co., Millis, MA, USA). The animals were ventilated for 15 minutes to stabilize the physiological parameters. According to the calculated dose of PFC for each group, the dose was divided into three equal aliquots that were administered at one-minute intervals through tracheotomy (*room temperature*) to achieve a homogeneous distribution. Following the administration of PFC, the animals were ventilated for 30 minutes, and the heart-lung block was harvested for histological and morphometric analyses. The morphometry was based on the technique established by Weibel. [27], consisting of determining the number of times that the structures of the lung parenchyma intersect a set of straight lines.

Upon completion of the dose study, another 72 male Wistar rats weighing 250–300 g were used for the dose versus preservation time study. The animals were prepared for pulmonary perfusion as described elsewhere [28]. Preservation was performed using 20 mL of low-potassium dextran glucose preservation solution (LPD) at 4°C via antegrade cannulation of the pulmonary artery with drainage of the effluent via the left atrium. After flushing, the lungs were removed and randomized into 3 groups (*n* = 12): control in which the lungs were flushed with LPD; PFC 3 mL/kg (PFC 3), and PFC 7 mL/Kg (PFC 7) administered through tracheotomy at room temperature and perfused with the LPD solution. Each group was divided into four subgroups (*n* = 6) according to preservation time (3, 6, 12, and 24 hours).

Analysis of the variations of the thiobarbituric acid reactive substances (TBARS), determination of the caspase 3 and NF-*κ*B (subunit p65) activities, and TUNEL (terminal deoxynucleotidyl transferase-mediated dUTP nick-end labeling) staining in the lung grafts were used to assess the quality of lung preservation.

### 2.2. Orthotopic Left Lung Transplantation Study Groups

Orthotopic left lung transplantation was performed using the modified cuff technique we previously described [28]. Briefly, after general anesthesia with intraperitoneal ketamine (100 mg/kg) and xylazine (15 mg/kg), orotracheal intubation, mechanical ventilation, anticoagulation (300 U heparin), and median sternolaparotomy, the donor rat lungs were submitted to an antegrade flush via the pulmonary artery with 20 mL of cold (4°C) LPD solution at a pressure of 20 cm H_2_O. The heart-lung block was extracted with the lungs inflated at the end-tidal volume. The left lung graft was isolated, prepared, and stored in LPD at 4°C for 12 hours. The recipient animals were anesthetized, intubated (14-gauge catheter), and ventilated. They then underwent a left thoracotomy, and the pulmonary vessels and left bronchus were anastomosed using a standard cuff technique [9]. Transplants were performed to 6 animals per group, totaling 24 animals. Mean arterial pressure and arterial blood gases analysis were measured after 15 minutes in supine ventilation and at the end of the observation period.

In the lung transplant group (LTx group), transplantation was performed as described above. In the LTx/PFC group, the PFC at a dose of 7 mL/Kg was instilled endobronchially prior to the heart-lung harvest. After 12 hours of cold ischemia, the lung transplantation was performed in the same fashion as for the LTx group. The animals were euthanized after 120 minutes of reperfusion. Half of the left lung was removed and stored at −80°C, and the other half was stored in 10% formalin.

### 2.3. Oxidative Stress and Antioxidant Enzymes

The amount of aldehydes generated by lipid peroxidation was measured by the TBARS method, which measures the amount of substances reacting with thiobarbituric acid [29]. The analysis of superoxide dismutase (SOD) was based on the inhibition of the reaction of the superoxide radical with adrenalin [30]. The analysis of catalase (CAT) activity was based on measuring the decrease in hydrogen peroxide formation [31].

### 2.4. Caspase 3 Activity Assay and TUNEL Staining

Caspase 3 activity was analyzed as described by Forgiarini et al. [32] and TUNEL studies as described by Fischer et al. [13].

### 2.5. Western Blotting

Lung homogenates were prepared from frozen lungs (−80°C) and analyzed for the expression of the p65 subunit, caspase 3 and nitrotyrosine (Cell Signalling Biotechnology, Boston, MA, USA), and iNOS (Santa Cruz Biotechnology, Santa Cruz, CA, USA) by immunoblot analysis [33, 34].

### 2.6. Immunofluorescence

I*κ*B and caspase 3 immunofluorescence staining was performed on paraffin-embedded sections after deparaffinization in ethanol, xylene, and water. First, primary antibodies for polyclonal anti-I*κ*B and anti-caspase 3 (Cell Signaling Biotechnology, Boston, MA, USA) at 1 : 1000 were incubated overnight at 4°C. Cy3-conjugated goat anti-rat secondary antibody (Sigma Aldrich, USA) at 1 : 200 was incubated for 1 hour. Goat serum and the primary rat antibody were used as a negative control. Mounted slices were visualized within 2 hours on an Olympus Zeiss fluorescence microscope.

### 2.7. Lung Histology

Lung tissue specimens were fixed in formalin, dehydrated, cleared, and embedded in paraffin. Specimens were cut into 8 *μ*m serial sections and stained with hematoxylin eosin. One pathologist blinded to the experimental protocol and the region of sampling performed quantitative analysis by light microscopy. Each sample was examined under low and high power fields. At least four sections were obtained from each block, and 20 fields were randomly selected and analyzed for each section. The severity of the histological lesions was assessed using a score (HIS: histological score) [35].

### 2.8. Statistical Analysis

Data were expressed as means ± standard deviation. One-way analysis of variance (ANOVA) was used to determine the statistical significance. A value of *P* < 0.05 was considered statistically significant. When statistical significance was reached, it was followed by a post hoc analysis using the Student-Newman-Keuls test. Nonparametric data were analyzed using two-tailed unpaired *t*-tests. The data were analyzed using Statistical Package for Social Science version 17.0 statistical software (SPSS Inc., Chicago, IL, USA).

## 3. Results

A reduction in linear intercepts was found when the PFC7 and PFC15 lungs were compared with control lungs (*P* < 0.05). However, the PFC15 lungs showed a more pronounced reduction in the number of crossed septae compared with the PFC3 and PFC7 lungs (*P* < 0.05). Additionally, because all PFC15 lungs presented rupture of alveolar septae due to overdistention, we decided to exclude the 15 mL/kg dose experiments.

**Figure 1:**
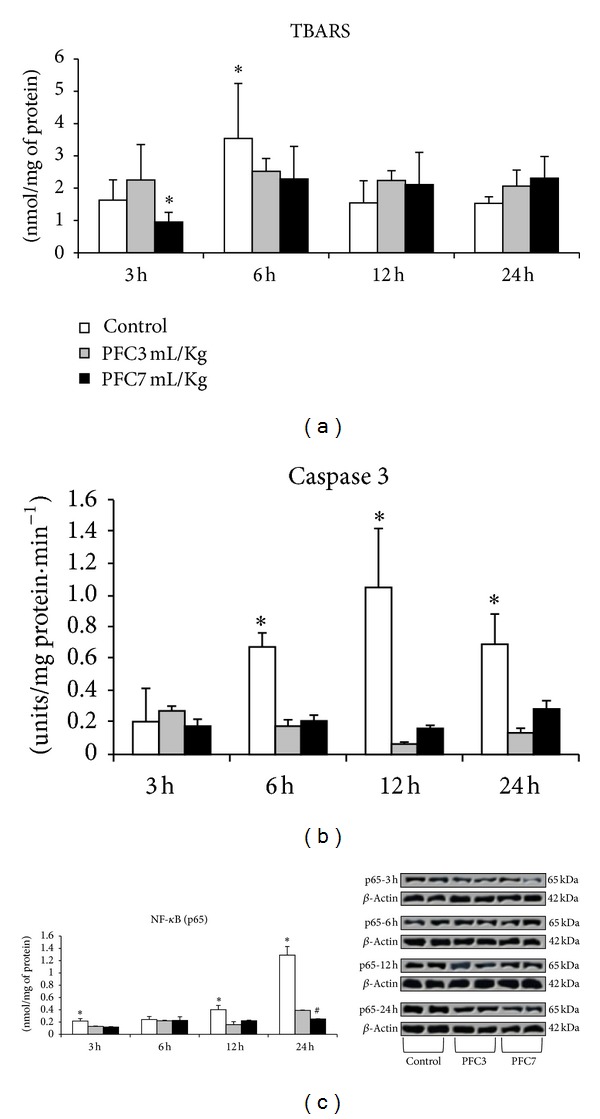
Apoptosis and inflammatory activity during lung preservation. (a) Effects of different periods of lung preservation on pulmonary lipid peroxidation. Using the TBARS assay, there was a significant decrease in lipid peroxidation at 3 hours of preservation in the PFC7 group (*P > 0.05). The control group showed a significant increase in lipid peroxidation at 6 h (^#^ *P* > 0.05). Data are means ± standard deviation of the mean (*P* > 0.05). (b) Caspase 3 activity at different times of lung preservation. There is a significant increase in caspase 3 activity in the control group at 6, 12, and 24 hours (*P < 0.001). Data are means ± SD of the mean. (c) NF-*κ*B expression (subunit p65) in the lung tissue at different times of lung preservation. A significant increase in the expression of p65 was observed in the control group at 3, 12, and 24 hours compared with the animals that used PFC (*P < 0.05). PFC7 reduced the expression of p65 at 24 hours of preservation compared with PFC3 (^#^ *P* < 0.05).

**Figure 2:**
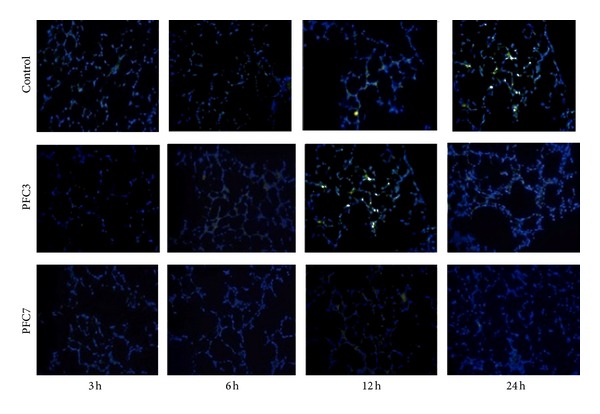
TUNEL staining after the different periods of cold ischemia. DAPI and FITC staining: DAPI-positive cells (nucleated cells) are blue and FITC-positive cells (TUNEL-positive) are green. There is predominance of TUNEL-positive cells in PFC 3 group at 12 h of cold preservation.

When lipid peroxidation was evaluated at the different lung preservation periods, there were significant differences between the PCF3 and PFC7 groups in the quantity of lipid peroxidation products at three hours of preservation (*P* < 0.05). At six hours, we observed a significant increase in TBARS in the control group compared with PFC3 and PFC7 (*P* < 0.05). However, at 24 hours of preservation, there were no differences between the groups with and without PFC (Figure 1). When apoptosis was measured by caspase 3 activity, a significant increase in the lungs of the control groups was observed compared with the groups using PFC for lung preservation in the lungs submitted to 6, 12, and 24 hours of storage (*P* < 0.05) (Figure 1). The TUNEL staining showed the presence of more FITC-positive cells (TUNEL-positive) at 3 and 12 hours of preservation (Figure 2).

The expression of NF-*κ*B was significantly reduced at 3, 12, and 24 hours of preservation in the PFC lungs compared with the control lungs (*P* < 0.05). However, after 24 hours of lung preservation, a significant reduction in the expression of NF-*κ*B was found in the PFC7 group compared with the lungs in the PFC3 group (*P* < 0.05) (Figure 1).

After transplantation, there were no differences in lipid peroxidation and antioxidant enzymes between the PFC and control groups in both the TBARS and catalase assays. The activity of superoxide dismutase was significantly higher in the LTx/PFC group compared with the LTx group (*P* < 0.05).

**Figure 3:**
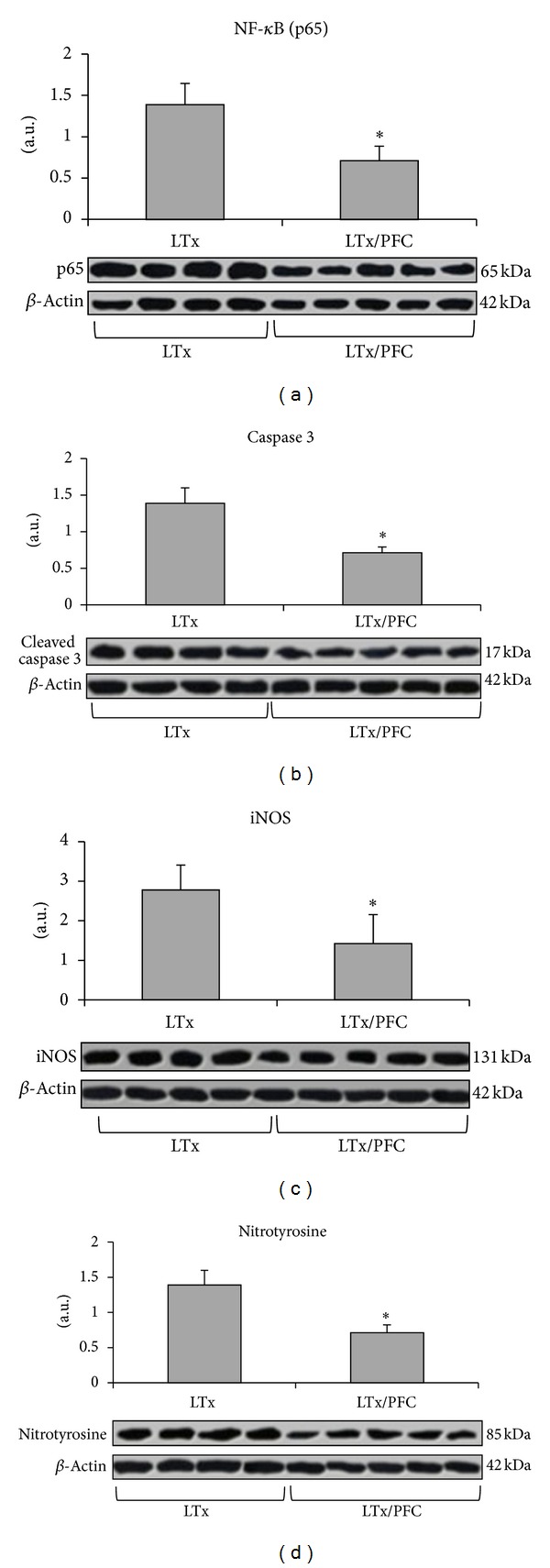
Apoptosis and inflammatory activity after lung transplantation. (a) Expression of NF-*κ*B (subunit p65) in the lung tissue after transplantation. There was an increased expression of NF-*κ*B subunit p65 in the LTx group compared with the LTx/PFC group (*P < 0.05). The images are representative samples of the groups. (b) Caspase 3 expression in the lung tissue after transplantation. There was a significant reduction in the expression of caspase 3 in the LTx/PFC group (*P < 0.05) compared with the LTx group. The images are representative samples of the groups. (c) Effect of lung transplantation on iNOS activity. iNOS activity was significantly higher in the LTx group (*P < 0.05). These results indicate that LTx induced the overexpression of iNOS, and the PFC treatment reduced iNOS expression. The images are representative samples of the groups. (d) Expression of nitrotyrosine in the lung tissue after transplantation. Nitrotyrosine expression was significantly lower in the LTx/PFC group compared with the LTx group (*P < 0.05). The images are representative samples of the groups.

**Figure 4:**
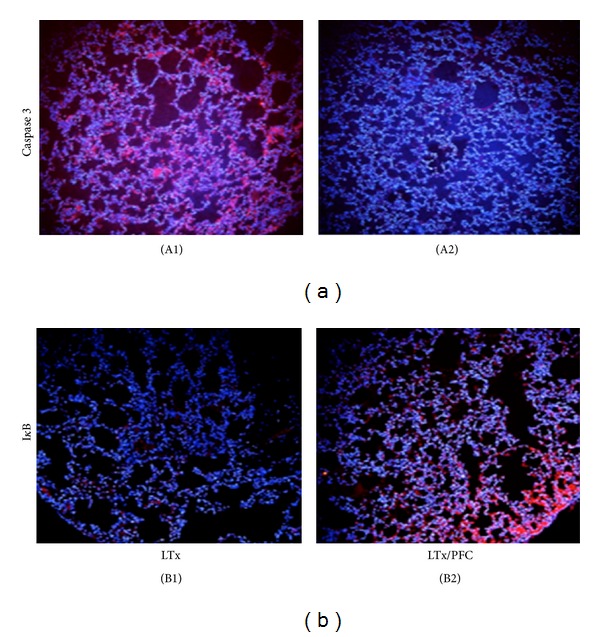
Immunofluorescence staining of caspase 3 (a) and I*κ*B (b). Cy3-positive cells (nucleated cells) are in red. There was an increased number of apoptotic cells by caspase 3 staining in the LTx group (A1) and an inhibition of phosphorylation of the inhibitor I*κ*B in the LTx/PFC group (B2). ((A1) and (B1)—LTx group; (A2) and (B2)—LTx/PFC group.)

The partial pressure of arterial oxygen (PaO_2_), carbon dioxide (PaCO_2_), and mean arterial pressure (MAP) did not show any significant differences between both groups at the different time points (Table 1). After lung transplantation, there was a significant increase in the nuclear translocation of NF-*κ*B (subunit p65) in the LTx group compared with the LTx/PFC group (*P* < 0.05) (Figure 3). There was also a significant increase in iNOS expression and caspase 3 expression in the LTx group (*P* < 0.05) compared with the LTx/PFC group (Figure 3). The same pattern was observed for nitrotyrosine (Figure 3). When we evaluated apoptosis by immunofluorescence and the inhibition of NF-*κ*B by its inhibitor I*κ*B, we observed an increase in caspase 3 and a reduction in I*κ*B in the LTx group. However, the lungs subjected to preservation with liquid PFC showed a decrease in caspase 3 and inhibition of phosphorylation of I*κ*B (Figure 4).

**Figure 5:**
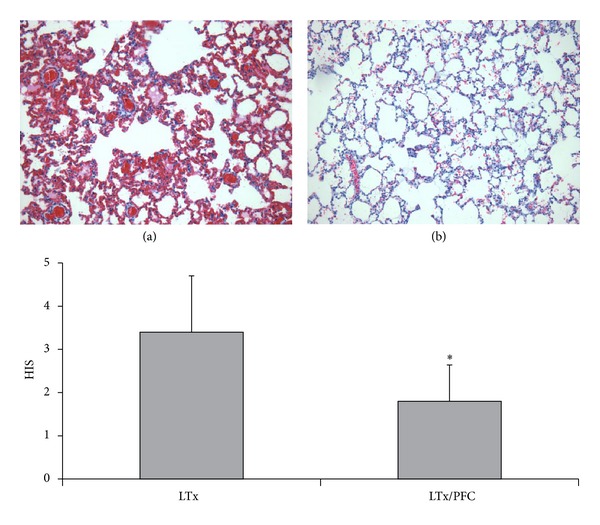
Histological score (HIS) after the lung transplantation. Lung injury was characterized by perivascular edema, intra-alveolar hemorrhage, and interstitial and intra-alveolar leukocyte infiltration in the LTx group (a) compared with the LTx/PFC group (b). The HIS demonstrated significantly more lung injury in the Ltx group (*P < 0.05) (200x magnification).

The histopathology of the lungs in the LTx group showed severe changes in the lung morphology characterized by cellular infiltrates, thickening of the alveolar septae, and atelectasis, which were not observed in the LTx/PFC group. The analysis of the severity of the histological damage using the histology score (HIS) showed a significant reduction in lung injury in the LTx/PFC group compared with the LTx group (Figure 5).

## 4. Discussion

Our results showed that liquid perfluorocarbon administered endobronchially was able to improve lung preservation and reduced the deleterious effects of IR injury. To our knowledge, this study is the first report of such benefits on lung preservation in this experimental model of lung transplantation.

Initially, the PFC was administered into the airways to enhance graft preservation and evaluate two different PFC doses at four distinct cold preservation times. Both PFC doses (3 and 7 mL/kg) were able to reduce cell apoptosis and the inflammatory response for up to 12 hours of preservation compared with the control group. Our study differs from Fischer et al.'s study [13], which showed almost no apoptosis and cell death in rat lungs preserved for 12 h. However, these authors showed a significant increase in cell death at 18 and 24 h of preservation, which was similar to our findings. This slight variability may derive from the different techniques employed to demonstrate apoptosis in both studies.

In our study, the PFC yielded a significant reduction in caspase 3 activity up to 24 h, which is an indicator that PFC may provide an additional protection to the grafts. Additionally, we observed a significant decrease in the TUNEL-positive cells at 7 mL/kg, showing fewer necrotic cells at 24 hours.

Another important player during graft preservation is the presence of NF-*κ*B, an important rapid-response transcription factor that is critical to the regulation of apoptosis and has been shown to trigger the inflammatory response after transplantation [8, 14] through the dissociation of the complex with I*κ*B-a releasing p65. Our study showed that 7 mL/kg PFC significantly reduced p65 activity compared with 3 mL/kg at 24 hours. This finding enabled us to define 7 mL/kg as the ideal dose to confer longer graft preservation with less inflammatory activity.

In our experimental lung transplantation model, we used lungs preserved for 12 hours, which corresponded to the highest expression of apoptotic cells in the control group. This finding would represent potentially reversible cell activity.

Perfluorocarbon has a protective role in the surfactant system and alveolar membrane [36]. Although PFC has a high oxygen and CO_2_ diffusion capacity, we did not observe any improvement in gas exchange after lung transplantation in both groups. These findings can be related to the ischemic time because, at 12 hours, the number of necrotic cells was not sufficient to induce detectable physiological changes. Additionally, the contralateral lung could be responsible for maintaining adequate gas exchange, since it was not excluded in our model. Furthermore, in this model of lung transplantation, the MAP was similar in both groups, demonstrating that PFC did not lead to any hemodynamic imbalance in the posttransplantation period.

The assessment of oxidative stress by catalase and TBARS was not sensitive enough in our model. Other tests such as isoprostane quantification may be able to demonstrate much earlier changes in this setting. Similar results have been found in a previous study by Pilla et al. [10] in an animal model of ischemic preconditioning. However, studies that used different techniques have found significant differences in oxidative stress in a similar animal model of lung transplantation [9, 37]. Lastly, endobronchial PFC has been shown to protect lungs against reactive oxygen species after lung transplantation, as demonstrated by an increase in superoxide dismutase [38].

Lungs transplanted after longer preservation periods have severe IR injury, a significant lung functional impairment, and an increased number of dead cells [21]. The suppression of caspase activity has been shown to correlate with a decrease in apoptosis [13]. Our study demonstrated that PFC can significantly suppress the expression of caspase 3, thereby suggesting that there is a reduction in cell death. Quadri et al. [39] demonstrated that the beginning of the cell death process seems to occur at a much earlier stage of IR injury than previously thought. Caspases 3 and 8 had significantly increased activities immediately after the lungs were flushed with LPD. Such findings suggest that mechanisms underlying IR injury as well as programmed cell death start quite early in IR injury and continue to affect the intracellular processes even during the cold ischemic storage.

In our study, PFC significantly reduced iNOS expression after transplantation, indicating its anti-inflammatory and lung protective properties against IR. In addition, the chemical and physical properties of PFC confer additional protection to the lungs, preventing alveolar collapse and preserving the alveolar structure. Recently, using a model of ischemia reperfusion injury [32], in order to avoid the interference of variables that could have influenced results in a model of lung transplantation, we have shown that the use of endobronchial PFC reduced the inflammatory response, preserved the alveolar structure, and protected the lungs against the hazardous effects of IR injury [40].

The rationale for using PFC is mostly to protect the lungs once lung injury has been established [19, 23, 26, 40]. Based on such observations and the results of the present study, which suggests that the PFC can also be used before the onset of severe lung injury, lung transplantation can be a suitable model in which the PFC can provide both graft protection and IR injury reduction.

We conclude that, in the present animal model, the use of endobronchial PFC as an adjuvant to the current preservation strategy has improved graft preservation and reduced the hazardous effects of IR injury following lung transplantation. Future studies in a larger animal model are necessary to confirm the present findings.

## Figures and Tables

**Table 1:** Hemodynamics and gas exchange in the transplantation groups.

Groups	MAP		*P*	PaO_2_		*P*	PaCO_2_		*P*
	Initial	Final		Initial	Final		Initial	Final	
LTx	61.7 ± 2.6	41.1 ± 19.8	0.227	140.2 ± 30.6	130.9 ± 46.7	0.691	38.5 ± 13.7	37.3 ± 14.8	0.887
LTx/PFC	76.8 ± 11.8	60.4 ± 18.8	0.10	124.4 ± 32.1	113 ± 36.7	0.579	34.4 ± 6	38.1 ± 7.9	0.382

Values are means ± standard deviation.

LTx: lung transplant (control); LTx/PFC: lung transplant + perfluorocarbon; MAP: mean arterial pressure; PaCO_2_: partial pressure of arterial carbon dioxide; PaO_2_: partial pressure of arterial oxygen.
